# Putting the House in Order

**Published:** 1891-05-23

**Authors:** 


### Passing Topics.

### PUTTING THE HOUSE IN ORDER.

The attention which our hospitals have lately attracted will not be accounted altogether useless if it prove to have helped to strengthen their managers' sense of responsibility. We are conscious of a healthy vibration through the hospital system, and no worthy institution, as it seems to us, need waste a single regret upon the publicity given to its affairs, even though certain defects are thereby laid bare, for is it not the better enabled to apply a remedy?

Of course it is a misfortune when the tendency is to administer untempered criticism, but the hospital body is robust enough, upon the whole, to be more or less independent of climatic influences, and we are sanguine enough to believe that the time is not distant when deserving institutions will be the better and the stronger for the ordeal they have been called upon to endure.

Among the most important signs of awakened responsibility are the efforts of several hospitals to bring their sanitary arrangements up to date, and the authorities are undoubtedly entitled to credit for the energies they are expending in this direction. It will, perhaps, be said that in one notable instance the hospital is only moving under compulsion, but even if that be true, the same cannot be alleged of the London, St. George's, and the Victoria Park Consumption Hospital, where the movement has been entirely voluntary.

Hospitals depending on, and insufficiently supported by, charitable gifts often undergo hardships, and among the rest we may reckon the fact that they are expected to make their precarious incomes suffice not only for the day by day requirements, but for permanent works fairly chargeable to capital, assuming them to possess any. St. George's, we believe, has been fortunate enough of late years to be enabled by means of bequests to add largely to its reserves, and the latter are doubtless ample to cover the contemplated expenditure, heavy though it be. In the case of the smaller institution at Victoria Park, it is proposed to raise a special fund of £10,000, and without a grain of the sarcasm usually comprehended in the aspiration, we devoutly hope they may get it. The object in view is not only commendable; it is indispensably necessary. Obviously, when so great advances have been made in sanitary science, hospital buildings which have been in use for, say, no more than a couple of decades need overhauling, and the older hospitals must be altogether faulty.

Something to this effect appeared in these columns long before the present movement set in, and when the impeachment was far from popular. We are entirely upon the side of the experts, but not less because we believe these authorities are right do we strenuously protest against the notion that blame attaches to needy institutions for not having sooner incurred an expenditure which they have no funds to meet. Let the public find the means, and then they may fairly insist upon much which under present conditions is impracticable. Individuals depending upon a daily wage, and who live from hand to mouth, are not usually found ready to enter with alacrity upon permanent improvements, and starving hospitals are often too solicitous about their daily bread to concern themselves deeply with less pressing needs.

Agricola.

